# Anti-Inflammatory Activity of Tanzawaic Acid Derivatives from a Marine-Derived Fungus *Penicillium steckii* 108YD142

**DOI:** 10.3390/md14010014

**Published:** 2016-01-08

**Authors:** Hee Jae Shin, Gam Bang Pil, Soo-Jin Heo, Hyi-Seung Lee, Jong Seok Lee, Yeon-Ju Lee, Jihoon Lee, Ho Shik Won

**Affiliations:** 1 Marine Natural Products Chemistry Laboratory, Korea Institute of Ocean Science and Technology, 787 Haeanro, Ansan 426-744, Korea; vlfrkaqkd@kiost.ac (G.B.P.); hslee@kiost.ac (H.-S.L.); jslee@kiost.ac (J.S.L.); yjlee@kiost.ac (Y.-J.L.); jihoonlee@kiost.ac (J.H.L.); 2 Department of Applied Chemistry, Hanyang University, 55 Hanyangdaehak-ro, Sangnok-ku, Ansan, Gyeongi-do 15588, Korea; hswon@hanyang.ac.kr; 3 Jeju International Marine Science Center for Research & Education, Korea Institute of Ocean Science and Technology, Jeju 63349, Korea; sjheo@kiost.ac.kr

**Keywords:** marine fungus, *Penicillium steckii*, tanzawaic acids, anti-inflammatory

## Abstract

Chemical investigation of a marine-derived fungus, *Penicillium steckii* 108YD142, resulted in the discovery of a new tanzawaic acid derivative, tanzawaic acid Q (**1**), together with four known analogues, tanzawaic acids A (**2**), C (**3**), D (**4**), and K (**5**). The structures of tanzawaic acid derivatives **1**–**5** were determined by the detailed analysis of 1D, 2D NMR and LC-MS data, along with chemical methods and literature data analysis. These compounds significantly inhibited nitric oxide (NO) production and the new tanzawaic acid Q (**1**) inhibited the lipopolysaccharide (LPS)-induced inducible nitric oxide synthase (iNOS) and cyclooxygenase-2 (COX-2) proteins and mRNA expressions in RAW 264.7 macrophages. Additionally, compound **1** reduced the mRNA levels of inflammatory cytokines. Taken together, the results of this study demonstrated that the new tanzawaic acid derivative inhibits LPS-induced inflammation. This is the first report on the anti-inflammatory activity of tanzawaic acid Q (**1**).

## 1. Introduction

Although the nature of the associations between sponges and fungi is far from understood, there is accumulating evidence that some sponge-associated fungi have adopted the ability to produce chemical compounds which are structurally diverged from their terrestrial counterparts [1]. Marine sponge-associated fungi often live in sponges and their bioactive metabolites may be interpreted as chemically mediated defense mechanisms for protecting their host organism from environmental dangers such as predation [2]. Many studies have reported on the isolation of marine sponge-associated *Penicillium* spp. as producers of bioactive metabolites. Meanwhile, a growing number of studies have indicated that marine fungi can provide sources of novel bioactive secondary metabolites [3,4] and produce the decalin moiety-containing secondary metabolites. Frequently, they are highly functionalized through the substitution of methyl, hydroxyl, or C=C and C=O double bonds on the decalin skeleton, or through a three-, five-, or seven-membered side chain with carboxyls (or its ester), several double bonds, or via ring formation [5]. Their intricate structures and diverse biological activities have attracted researchers around the world to investigate their biosynthesis, chemical synthesis, and the various facets of the functionalized decalin skeleton [5].

**Figure 1:**
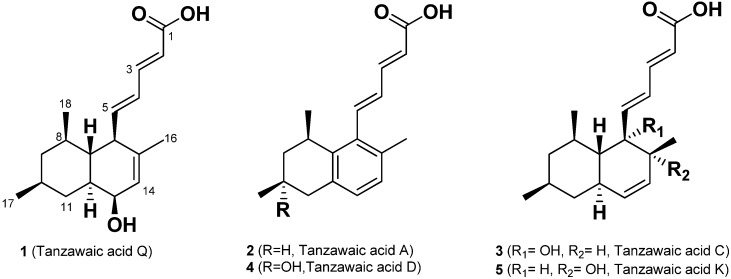
The structures of **1**–**5** isolated from the extract of *Penicillium steckii* 108YD142.

Inflammation is a normal immune process to protect the body from infection or tissue injury. During the inflammatory process, the stimulated immune monocytes and macrophages overexpress inducible nitric oxide synthase (iNOS) and cyclooxygenase-2 (COX-2) and secrete increased amounts of inflammatory mediators such as nitric oxide (NO), prostaglandin E2 (PGE2), and interleukin-1β (IL-1β), interleukin-6 (IL-6), tumor necrosis factor-α (TNF-α). Overproduction of these factors is involved in cell damage and inflammatory disease [6]. Thus, inhibition of the production of these inflammatory mediators is an important target in the treatment of inflammatory diseases [7]. In our continuous search for bioactive compounds, we isolated a new anti-inflammatory tanzawaic acid Q (**1**) and four known tanzawaic acids A (**2**), C (**3**), D (**4**) and K (**5**), which have a decalin motif, from a marine sponge–associated fungus, *Penicillium steckii* 108YD142 (Figure 1). Here, we report the isolation, structure determination and anti-inflammatory activities of tanzawaic acid Q (**1**).

## 2. Results and Discussion

The fungal strain 108YD142 was isolated from a marine sponge sample collected at Wangdolcho, in the Republic of Korea’s Eastern reef, and identified as *Penicillium steckii* by 18s rRNA sequencing. The strain 108YD142 was cultured in Bennett’s medium at 28 °C for seven days. The culture broth was extracted with ethyl acetate and the crude extract was purified by flash open chromatography and reversed phase high-performance liquid chromatography (HPLC). The four known compounds were identified as tanzawaic acids A (**2**), C (**3**), and D (**4**), K (**5**) by comparative analysis data of their NMR, MS and optical rotation values, indicating that these compounds share the same absolute configuration [8,9,10]. Compound **1** was isolated as yellow oil. The molecular formula was determined to be C_18_H_26_O_3_ based on the HR-ESIMS data *m*/*z* 313.1777 (calcd for [M + Na]^+^ 313.1780), indicating six indexes of unsaturation. The IR spectrum at 3397 and 1639 cm^−1^ represented the hydroxyl (OH) and carbonyl (CO) groups, respectively, and the UV spectrum showed a maximum absorption at 259 nm, suggesting that **1** is a tanzawaic acid derivative.

**Figure 2:**
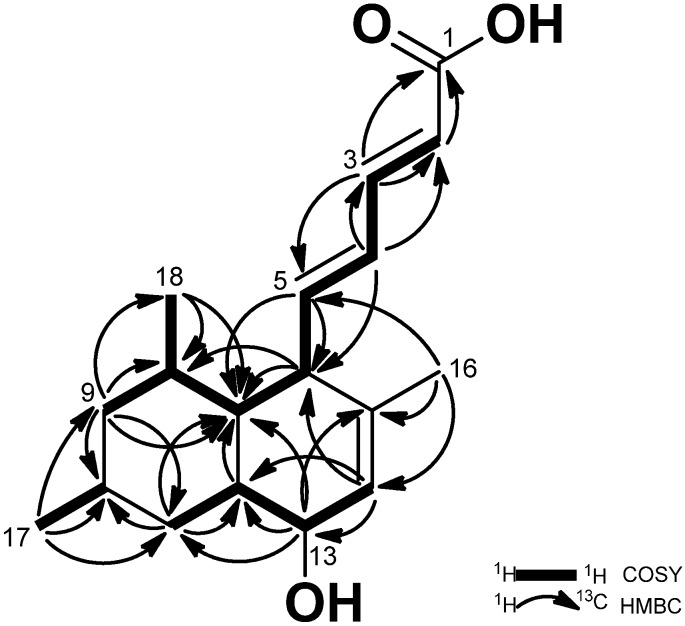
COSY and HMBC correlations of **1**.

The structure of **1** was elucidated by detailed analysis of 1D and 2D NMR data. The ^1^H NMR spectrum (Table 1) showed signals of five olefinic protons at δ_H_ 5.80–7.23, an oxymethine proton at δ_H_ 3.80, three methyl signals at δ_H_ 0.92, 0.94, 1.60, and nine aliphatic protons at δ_H_ 0.74–2.59. The ^13^C NMR spectrum (Table 1) showed 17 signals, which were identified as 11 methines, two methylenes, three methyls, and a quaternary carbon. However, the chemical shift of C-1 (δc 172.0) was obtained from the HMBC spectrum because the C-1 signal was not observed in the ^13^C NMR spectrum. The COSY spectrum appeared with four spin systems within the molecule starting from H-2 to H-9, H-8 to H-17, H-10 to H-18, and H-11 to H-14. The connectivity of the olefinic proton signals was confirmed by COSY correlations from H-2 (δ_H_ 5.83) to H-5 (δ_H_ 2.59). The hydroxyl group was located at C-13 by judging the chemical shifts of the oxygenated methine (δ_H_ 3.80, δ_C_ 68.6). The position of three methyl groups was determined at C-8, C-10, and C-15 by the HMBC correlations (Figure 2): H-18 (0.94)/C-7 (δ_c_ 44.8), C-8 (δ_c_ 41.6), C-9 (δ_c_ 47.1); H-17 (δ_H_ 0.92)/C-9 (δ_c_ 47.1), C-10 (δ_c_ 33.6), C-11 (δ_c_ 39.8); H-16 (δ_H_ 1.60, s)/C-6 (δ_c_ 51.9), C-14 (δ_c_ 127.1), C-15 (δ_c_ 139.8).

**Table 1:** ^1^H and ^13^C NMR data ^a^ of tanzawaic acid Q (**1**) in CD_3_OD.

Position	δ_C_, Type	δ_H_, Mult. (*J* in Hz)	HMBC	Key NOESY
1	172.0, qC ^b^			
2	122.2, CH	5.83, d (15.3)	1, 4, 5	
3	145.8, CH	7.23, dd (15.3, 11)	1, 2, 4, 5	
4	131.4, CH	6.31, dd (15, 11)	2, 3, 5	6
5	150.4, CH	6.01, dd (15, 9.5)	6, 7	7, 18
6	51.9, CH	2.59, t (7.5)	4, 5, 7, 8, 15	8, 12, 16
7	44.8, CH	1.29 ^c^	8	9_ax_, 11_ax_, 18
8	41.6, CH	1.35, brs		10
9	47.1, CH_2_	0.74_ax_, q (12)/1.58_eq_^c^	7, 8, 10, 11, 17	
10	33.6, CH	1.52 ^c^		
11	39.8, CH_2_	1.14_ax_, q (12)/1.55_eq_^c^	10, 12	
12	44.5, CH	1.29 ^c^	7	6, 8, 13
13	68.6, CH	3.80, d (6.5)	8, 12, 14, 15	11_eq_, 12, 14
14	127.1, CH	5.80, d (6.5)	6, 12, 13, 16	16
15	139.8, qC			
16	22.7, CH_3_	1.60, s	6, 14, 15	
17	23.2, CH_3_	0.92, d (6.5)	9, 10, 11	9_eq_
18	23.0, CH_3_	0.94, d (6)	7, 8, 9	9_ax_

^a^ ^1^H and ^13^C NMR data of **1** were measured at 500 and 125 MHz, respectively; ^b^ The chemical shift was obtained from the HMBC spectrum; ^c^ overlapping signals.

**Figure 3:**
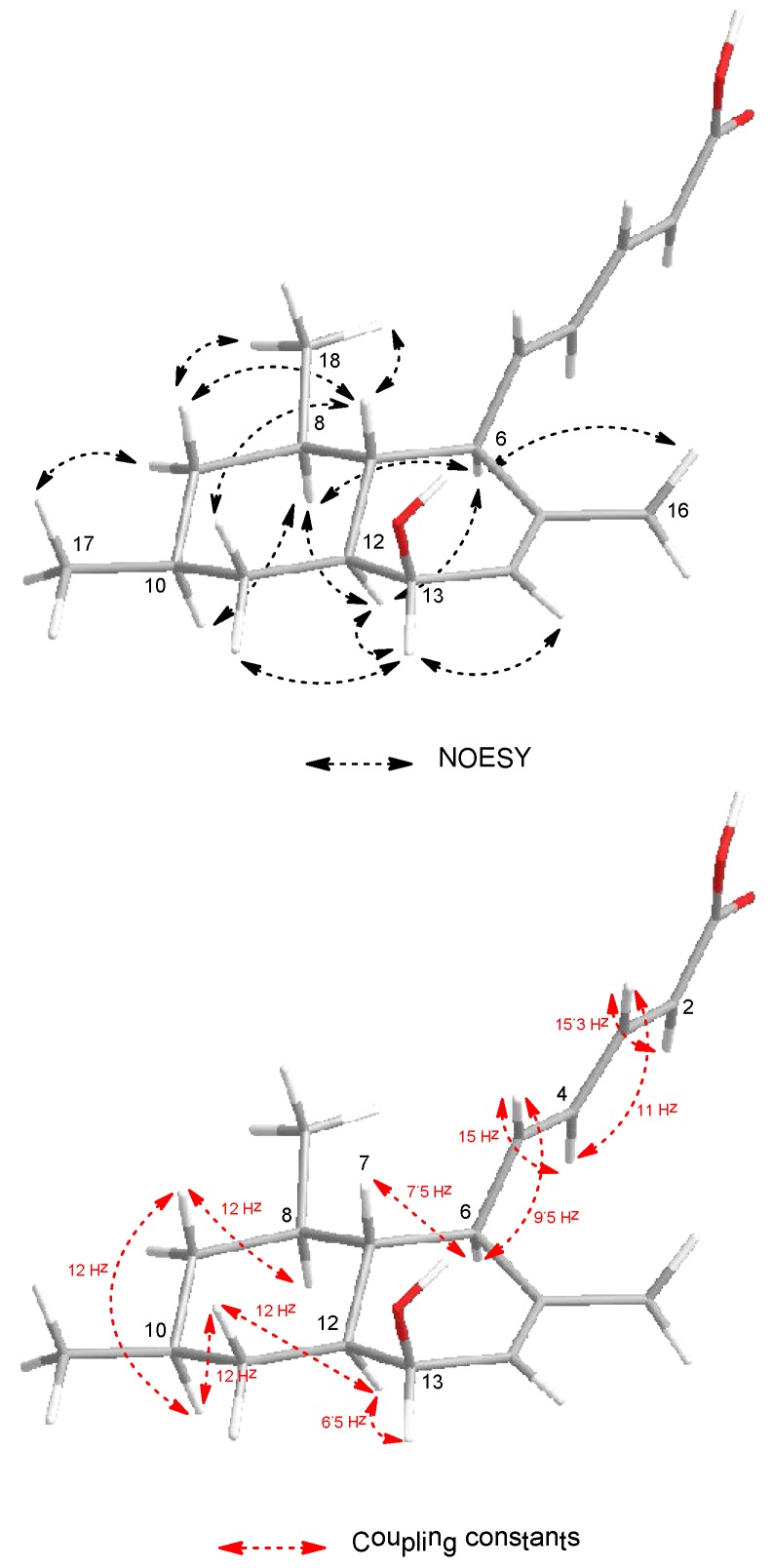
Selected NOESY correlations and coupling constants of **1**.

The configuration of the olefinic double bonds was deduced as *2E* and *4E* on the basis of the large coupling constants of H-2/H-3 (*J* = 15.3 Hz) and H-4/H-5 (*J* = 15 Hz). The relative configuration of **1** was determined by the NOESY spectrum and vicinal ^1^H–^1^H coupling constants (^3^*J*_vic_). NOESY correlations (Figure 3) of H-6 (δ_H_ 2.59) with H-8 (δ_H_ 1.35) and H-12 (δ_H_ 1.29) indicated a *trans* fusion of the rings. The axial orientations of H-7 (δ_H_ 1.29), H-8 (δ_H_ 1.35), H-9ax (δ_H_ 0.74), H-10 (δ_H_ 1.52), H-11ax (δ_H_ 1.14), H-12 (δ_H_ 1.29) and H-13 (δ_H_ 3.80) were indicated by the large coupling constants (^3^*J*_H-7,H-6_ = 7.5 Hz, ^3^*J*_H-9ax,H-8_ = ^3^*J*_H-9ax,H-10_ = ^3^*J*_H-11ax,H-10_ = ^3^*J*_H-11ax,H-12_ = 12 Hz, ^3^*J*_H-5,H-6_ = 9.5 Hz). H-13 showed only a small coupling (*J* = 6.5 Hz) to H-12 and was assigned to an equatorial orientation, indicating that the hydroxyl group at C-13 is located in an axial position. H-16, H-17, and the olefinic side chain were on the same side of the molecule. The NOESY correlation between the ring junction protons H-7 and H-12 was not observed, indicating their *anti* relationship. Thus, the relative configurations at C-6, C-7, C-8, C-10, C-12 and C-13 were established as shown in Figure 3.

The structure of compound **1** was found quite similar to the reported tanzawaic acid I identified from the culture filtrates of the fungal strain *Penicillium* sp. IBWF104-06 isolated from a soil sample [10]. The only difference was that compound **1** has a hydroxyl group at C-13 but tanzawaic acid I has one more hydroxyl group at C-6. The relative configuration of **1** was also similar to tanzawaic acid I. Thus, the structure of **1** was determined as a new tanzawaic acid derivative, named tanzawaic acid Q (**1**).

**Figure 4:**
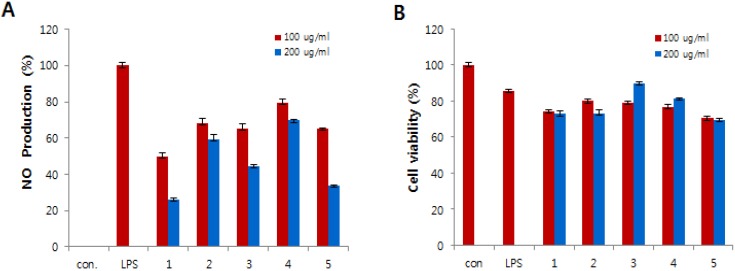
Effects of compounds **1**–**5** on nitric oxide (NO) production (**A**) and cell viability (**B**) in lipopolysaccharide (LPS)-induced RAW 264.7 macrophages. (**A**) The production of NO was measured in the culture medium of cells stimulated with LPS (1 μg/mL) for 24 h in the presence of compounds **1**–**5**. (**B**) Cytotoxicity of compounds **1**–**5** was assayed by the MTT method. Cells (1.5 × 10^5^ cells/mL) were pre-incubated for 16 h, and then cells were stimulated with LPS (1 μg/mL) in the presence of compounds **1**–**5** at the indicated concentrations for 24 h.

Compounds **1**–**5** were tested for *in vitro* anti-inflammatory activity by suppressing the production of NO in lipopolysaccharide (LPS)-induced RAW 264.7 cells. The RAW 264.7 cells were treated for 24 h with various concentrations of compounds **1**–**5** and cytotoxicity was estimated using MTT assay. All compounds exhibited less cytotoxic effect on cells in comparison to control cells (Figure 4B). LPS was used to stimulate the release of the inflammatory mediator NO from the RAW 264.7 macrophage cell line. The NO production was estimated from the accumulation of nitrite in the medium using the Griess reagent. As shown in Figure 4A, the production of NO was strongly inhibited by compounds **1**, **3**, and **5**.

**Figure 5:**
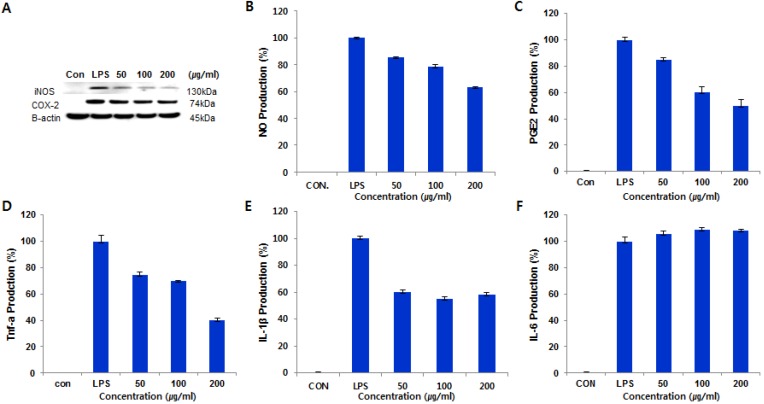
Cells (1.0 × 10^6^ cells/mL) were pre-incubated for 18 h, and then cells were stimulated with LPS (1 μg/mL) in the presence of compound **1** at the indicated concentrations for 24 h. (**A**) The protein levels of iNOS and COX-2 were determined by immunoblot analysis. The β-actin was used as a loading control. The effect of compound **1** on the production of NO (**B**); TNF-α (**C**); PGE2 (**D**); IL-1β (**E**); and IL-6 (**F**) in LPS-stimulated RAW 264.7 murine macrophage.

Based on this result, we studied further to investigate the effects of **1** on iNOS and COX-2 proteins and mRNA expressions in LPS-stimulated RAW 264.7 cells using immunoblot analysis. As shown in Figure 5A, compound **1** inhibited iNOS and COX-2 protein levels in a dose-dependent manner, while the house-keeping protein *β*-actin was unchanged by the existence of **1** at the same conditions. In addition, we measured the levels of inflammatory mediators, such as NO, PGE_2_ and TNF-α, IL-1β, and IL-6, in LPS-stimulated RAW 264.7 cells. Compound **1** significantly reduced the production of NO, TNF-α, and IL-1β, but did not affect the level of IL-6, as shown in Figure 5B–F.

Tanzawaic acid derivatives have been reported to have multiple biological activities [8,9,10,11,12,13]. Tanzawaic acid A (**2**) has been reported to have antimicrobial, anticoccidal activities and inhibited superoxide anion [8,9]. Tanzawaic acid K (**5**) has also antimicrobial and anti-proliferative activities [10]. The bioactivity tests revealed that tanzawaic acid C (**3**) has no cytotoxicity at the concentration of 200 μg/mL. Meanwhile, tanzawaic acid K (**5**) has moderate cytotoxic effect and inhibited NO production in LPS-stimulated RAW 264.7 murine macrophages at the concentration of 100 μg/mL. This inhibitory effect correlated with the inhibition effect of tanzawaic acid K (**5**) on iNOS expression in LPS-stimulated RAW 264.7 cells.

## 3. Experimental Section

### 3.1. General Experimental Procedures

The ^1^H, ^13^C, and COSY, NOESY, HSQC, HMBC NMR spectra were acquired on a Varian Unity 500 MHz spectrometer. Optical rotations were measured on a JASCO DIP-1000 digital polarimeter (JASCO Corporation, Tokyo, Japan), with a 1 cm cell. UV spectra were obtained on a Shimadzu UV-1650PC spectrophotometer (Shimadzu Corporation, Kyoto, Japan). IR spectra were recorded on a JASCO FT/IR-4100 spectrophotometer, (JASCO Corporation, Tokyo, Japan). High-resolution ESI mass spectroscopy was recorded on a hybrid ion-trap time-flight mass spectrometer (SYNAPT G2, Wasters Corporation, Milford, CT, USA). High performance liquid chromatography (HPLC) was conducted with a PrimeLine pump (Analytical Scientific Instrument, Inc., El Sobrante, CA, USA) with RI 2000 refractive index detector (JORDI Labs, Mansfield, UK). Semi-preparative HPLC was performed using ODS (YMC-pack-ODS-A, 250 × 10 mm i.d., 5 μm).

### 3.2. Isolation and Identification of the Strain 108YD142

The strain 108YD142 was isolated from a marine sponge sample collected at Wangdolcho, East Sea, Korea, in 2010, by serial dilution technique and grown on Bennett’s agar plates (1% glucose, 0.2% tryptone, 0.1% yeast extract, 0.1% beef extract, 0.5% glycerol, 1.7% agar, salinity 32 g/L, pH 7.0 before sterilization). The plates were incubated for seven days at 28 °C, and the resulting colony of the strain 108YD142 was isolated and stocked in 40% glycerol. According to BLAST and phylogenetic analysis based on 18s rRNA gene sequences, the strain was identified as *Penicillium steckii*. The sequence was deposited in GenBank under the accession number KU316936. This strain is currently preserved in the Microbial Culture Collection, Korea Institute of Ocean Science and Technology (KIOST), under the curatorship of Hee Jae Shin.

### 3.3. Seed and Mass Cultures of the Strain 108YD142

The seed and mass cultures of the strain 108YD142 were carried out in Bennett’s medium (1% glucose, 0.2% tryptone, 0.1% yeast extract, 0.1% beef extract, 0.5% glycerol, 3.2% sea salt, pH 7.0 before sterilization). The medium (300 mL) was provided into a 500 mL conical flask and sterilized. A single colony of the strain from the agar plate was inoculated into the flask and incubated at 28 °C for four days on a rotary shaker at 120 rpm. An aliquot (0.1% *v*/*v*) from the seed culture was inoculated into 2 L flasks (total 13 flasks) containing 1 L medium and a 20 L fermenter containing 17 L of sterilized medium. The mass culture was incubated under the same conditions as the seed culture for seven days and then harvested.

### 3.4. Extraction and Isolation of Compounds

The culture broth (total 30 L) was harvested by high speed centrifugation (60,000 rpm) and then extracted with EtOAc (two times). The EtOAc extract was evaporated to obtain crude extract (1.97 g). The crude extract was subjected to an ODS open column chromatography followed by stepwise gradient elution with MeOH/H_2_O (*v*/*v*) (1:4, 2:3, 3:2, 4:1, and 100:0) as eluent. The subfraction eluted with MeOH/H_2_O (4:1) was purified by a reversed-phase HPLC (YMC ODS-A column, 250 × 10 mm i.d, 5 μm; 70% MeOH in H_2_O; flow rate 1.5 mL/min; RI detector) to yield pure compounds **1** (1.7 mg, t*_R_* 115 min), **3** (4.2 mg, t*_R_* 88 min), **4** (12 mg, t*_R_* 42 min), and **5** (1.4 mg, t*_R_* 97 min) and the subfraction eluted with MeOH/H_2_O (100:0) was purified by a reversed-phase HPLC (YMC ODS-A column, 250 × 10 mm i.d., 5 μm; 90% MeOH in H_2_O; flow rate 1.5 mL/min; RI detector) to yield pure compound **2** (0.9 mg, t*_R_* 26 min).

*Tanzawaic acid Q* (**1**): Yellow oil; [α]${}_{D}^{26}$ + 69.5° (*c* 0.5, MeOH); UV (MeOH) λ_max_ (log ε) 259 (4.82); IR (MeOH) ν_max_ 3397, 2923, 1678, 1639, 1550, 1394, 1263 cm^−1^; ^1^H and ^13^C NMR data (Table 1); APCI-MS *m*/*z* 289.08 [M − H]^−^; HR-ESI-MS *m*/*z* 313.1777 [M + Na]^+^ (calcd for C_18_H_26_O_3_Na, *m*/*z* 313.1780 [M + Na]^+^).

*Tanzawaic acid A* (**2**): Yellow oil; C_18_H_22_O_2_; [α]${}_{D}^{24}$ + 297° (*c* 0.5, MeOH); UV (MeOH) λ_max_ (log ε) 205 (5.51); IR (MeOH) ν_max_ 3418, 2923, 1695, 1629 cm^−1^; APCI-MS *m*/*z* 268.94 [M − H]^−^.

*Tanzawaic acid C* (**3**): Yellow oil; C_18_H_26_O_3_; [α]${}_{D}^{24}$ + 173° (*c* 0.5, MeOH); UV (MeOH) λ_max_ (log ε) 258 (4.74); IR (MeOH) ν_max_ 3433, 2929, 1724, 1635, 1271 cm^−1^; APCI-MS *m*/*z* 289.06 [M − H]^−^.

*Tanzawaic acid D* (**4**): Yellow oil; C_18_H_22_O_3_; [α]${}_{D}^{30}$ + 56.4° (*c* 0.57, MeOH); UV (MeOH) λ_max_ (log ε) 207 (4.97); IR (MeOH) ν_max_ 3386, 2925, 1688, 1377, 1267 cm^−1^; APCI-MS *m*/*z* 284.65 [M − H]^−^.

*Tanzawaic acid K* (**5**): Yellow oil; C_18_H_26_O_3_; [α]${}_{D}^{26}$ − 29.1° (*c* 0.5, MeOH); UV (MeOH) λ_max_ (log ε) 257 (4.94); IR (MeOH) ν_max_ 3390, 2923, 1681, 1643, 1457, 1270 cm^−1^; APCI-MS *m*/*z* 289.03 [M − H]^−^.

### 3.5. Cell Culture

The murine macrophage cell line RAW 264.7 was purchased from the Korean Cell Line Bank (KCLB; Seoul, Korea). RAW 264.7 cells were cultured in Dulbecco’s modified Eagle’s medium (DMEM; GIBCO Inc., NY, USA) supplemented with 100 U/mL of penicillin, 100 μg/mL of streptomycin and 10% fetal bovine serum (FBS; GIBCO Inc., New York, NY, USA). The cells were incubated in a humidified atmosphere of 5% CO_2_ at 37 °C and sub-cultured every three days.

### 3.6. Determination of Cell Viability

Cell viability was measured twice using the conventional MTT assay. RAW 264.7 cells were seeded in 96-well plates at a density of 1.5 × 10^5^ cells/mL. After 16 h, the cells were treated with LPS (1 μg/mL) and samples, followed by additional incubation for 24 h at 37 °C. MTT stock solution (2 mg/mL) was added to wells for a total reaction volume of 200 μL. After 4 h incubation, the plates were centrifuged for 5 min at 800 g, and the supernatants were aspirated. The formazan crystals in each well were dissolved in 150 μL of DMSO, and the absorbance was measured using a microplate reader (ThermoMax, Sunnyvale, CA, USA) at a wavelength of 540 nm. Relative cell viability was evaluated based on the quantity of MTT converted to the insoluble formazan salt. The optical density of formazan generated in the control cells represented 100% viability. The data are expressed as mean percentages of the viable cells compared to the respective control.

### 3.7. Determination of NO Production

After pre-incubation of RAW 264.7 cells (1.5 × 10^5^ cells/mL) with LPS (1 μg/mL) and samples at 37 °C for 24 h, the quantity of nitrite accumulated in the culture medium was measured twice as an indicator of NO production [14]. Briefly, 100 μL of cell culture medium were mixed with 100 μL Griess reagent (1% sulfanilamide and 0.1% naphthylethylenediamine dihydrochloride in 2.5% phosphoric acid), and incubated at room temperature for 10 min. Absorbance at 540 nm was measured in a microplate reader (ThermoMax, Sunnyvale, CA, USA). Fresh culture medium was used as the blank in all determinations.

### 3.8. Determination of PGE_2_ Production

Samples were diluted with DMEM before treatment. Cells were treated with LPS (1 μg/mL) to allow cytokine production for 24 h. The PGE_2_ concentration in the culture medium was quantified using a competitive enzyme immunoassay kit (R & D Systems, Minneapolis, MN, USA) according to the manufacturer’s instructions. PGE_2_ production was measured relative to that of the control.

### 3.9. Measurement of Pro-Inflammatory Cytokine Production

Samples solubilized in DMSO were diluted with DMEM before treatment. The inhibitory effect of samples on pro-inflammatory cytokine (TNF-α, IL-1β, and IL-6) production from LPS (1 μg/mL)-treated RAW 264.7 cells was determined as described by Cho *et al.* [15]. Supernatants were assayed using mouse ELISA kits (R&D Systems Inc., Minneapolis, MN, USA).

## 4. Immunoblotting

RAW 264.7 cells (1.0 × 10^6^ cells/mL) were treated with LPS (1 μg/mL) and samples for 24 h, and cellular proteins were extracted from the cells. Protein concentrations were determined using a Bio-Rad protein assay kit (Bio-Rad, Hercules, CA, USA) with bovine serum albumin (BSA) as a standard. Cell lysates (30–50 μg) were separated by SDS–PAGE (8%–12%), and the separated proteins transferred to polyvinylidene difluoride (PVDF) membranes (Bio-Rad) for 2 h. The membranes were pre-incubated with blocking solution (5% skim milk in Tris-buffered saline containing Tween-20) at room temperature for 2 h and then incubated with anti-mouse iNOS (1:1000; Calbiochem, La Jolla, CA, USA) and anti-mouse COX-2 (1:1000; BD Biosciences Pharmingen, San Jose, CA, USA) for 2 h at room temperature. After washing, the blots were incubated with horseradish peroxidase conjugated goat anti-mouse IgG secondary antibody (1:5000; Amersham Pharmacia Biotech, Little Chalfont, UK) for 30 min. The bands were visualized by chemiluminescence on X-ray film using ECL detection reagent (Amersham Biosciences, Piscataway, NJ, USA).

## 5. Conclusions

As a result, we isolated a new tanzawaic acid derivative, tanzawaic acid Q (**1**), and four known analogues, tanzawaic acids A (**2**), C (**3**), D (**4**) and K (**5**), from a marine-derived fungus, *Penicillium steckii* 108YD142. Tanzawaic acids Q (**1**), C (**3**) and K (**5**) strongly inhibited LPS-induced NO production. Especially, tanzawaic acid Q (**1**) inhibited the expression of iNOS and COX-2 proteins in RAW264.7 cells. Also, **1** depleted the production of PGE_2_, TNF-α, and IL-1β mRNA protein. Based on the anti-inflammatory effects of tanzawaic acid Q (**1**), C (**3**) and K (**5**) against LPS-stimulated RAW 264.7 macrophages, it could be suggested that the position of the hydroxyl group (OH) or the presence of the carboxyl group (COOH) in the structure might be key structural features for the anti-inflammatory activity of tanzawaic acids. Tanzawaic acid A, which has an aromatic ring, exhibited good anti-inflammatory activity [6], suggesting that the aromatic ring is also important for the activity. In conclusion, this study revealed for the first time that tanzawaic acid Q (**1**) possesses anti-inflammatory activity. However, further studies are needed to know the detailed mechanism of the anti-inflammatory activity of **1**.
